# Identification of hypoxia‐related gene signatures based on multi‐omics analysis in lung adenocarcinoma

**DOI:** 10.1111/jcmm.18032

**Published:** 2023-11-27

**Authors:** Qineng Luo, Xing Li, Zixing Meng, Hao Rong, Yanguo Li, Guofang Zhao, Huangkai Zhu, Lvjun Cen, Qi Liao

**Affiliations:** ^1^ School of Public Health Health Science Center Ningbo University Ningbo Zhejiang P. R. China; ^2^ Department of Thoracic Surgery Hwa Mei Hospital University of Chinese Academy of Sciences Ningbo Zhejiang P. R. China; ^3^ The First Affiliated Hospital Ningbo University Ningbo Zhejiang P. R. China

**Keywords:** DNA methylation, gene expression, hypoxia, lung adenocarcinoma, multi‐omics, prognostic model, somatic mutation

## Abstract

Lung adenocarcinoma (LUAD) is the most common type of lung cancer and one of the malignancies with the highest incidence rate and mortality worldwide. Hypoxia is a typical feature of tumour microenvironment (TME), which affects the progression of LUAD from multiple molecular levels. However, the underlying molecular mechanisms behind LUAD hypoxia are not fully understood. In this study, we estimated the level of hypoxia by calculating a score based on 15 hypoxia genes. The hypoxia scores were relatively high in LUAD patients with poor prognosis and were bound up with tumour node metastasis (TNM) stage, tumour size, lymph node, age and gender. By comparison of high hypoxia score group and low hypoxia score group, 1820 differentially expressed genes were identified, among which up‐regulated genes were mainly about cell division and proliferation while down‐regulated genes were primarily involved in cilium‐related biological processes. Besides, LUAD patients with high hypoxia scores had higher frequencies of gene mutations, among which *TP53*, *TTN* and *MUC16* had the highest mutation rates. As for DNA methylation, 1015 differentially methylated probes‐related genes were found and may play potential roles in tumour‐related neurobiological processes and cell signal transduction. Finally, a prognostic model with 25 multi‐omics features was constructed and showed good predictive performance. The area under curve (AUC) values of 1‐, 3‐ and 5‐year survival reached 0.863, 0.826 and 0.846, respectively. Above all, our findings are helpful in understanding the impact and molecular mechanisms of hypoxia in LUAD.

## 1 INTRODUCTION

Lung cancer is still the main cause of cancer deaths, with an estimated 2.2 million new cancer cases (11.4%) and an estimated 1.8 million new deaths (18.0%) worldwide in 2020 according to the GLOBOCAN 2020 project.^1^, ^2^ The vast majority of lung cancer is non‐small cell lung cancer (NSCLC), accounting for 85% of primary lung cancer cases, in which adenocarcinoma is the most important histological type.^1^, ^3^ Currently, lung adenocarcinoma (LUAD) is treated with surgery, radiotherapy, chemotherapy, targeted therapy and immunotherapy or a combination of these therapies.^4^ However, the high tolerance of LUAD to conventional radiotherapy and chemotherapy agents still hinders the progress of treatment effectiveness. Acquired drug resistance has become common in the treatment of LUAD.^5^

Hypoxia is an important feature of tumour microenvironment (TME),^6^ which is closely related to cell proliferation,^7^ angiogenesis,^8^, ^9^ metabolism,^10^ metastasis,^11^, ^12^ tumorigenesis^13^ and tumour progression.^14^ Hypoxia affects the prognosis of patients by modulating TME and makes tumours resistant to conventional therapy.^15^ Intratumoral hypoxia could induce hypoxia‐inducible factor 1α (HIF‐1α) overexpressed in tumours, and there is a close relationship between tumour treatment failure and increased mortality.^16^ Therefore, hypoxia‐targeted therapy is promising for LUAD patients and may help overcome resistance in LUAD treatment.

Multi‐omics integration approaches can dissect tumours in multiple dimensions, analyse the response of tumour cells to chemotherapy and explore possible molecular features with potential diagnostic and prognostic significance.^17^ Therefore, to penetrate the complexity of LUAD, it is necessary to discover cancer prognostic biomarkers at systems level by comprehensively analysing multi‐omics resources to identify molecular changes.^18^

In the present study, we found hypoxia scores were obviously correlated with shorter overall survival in LUAD patients and a prognostic model for LUAD was established in view of 25 hypoxia‐related features from multi‐omics level. This study aims to explore the effect of hypoxia on LUAD from the perspective of multi‐omics by using bioinformatics methods, and to explore the potential molecular mechanisms behind hypoxia in LUAD. We hope this study could provide some predictive features with clinical application value for LUAD patients' early diagnosis and prompt treatment.

## 2 MATERIALS AND METHODS

### 2.1 Data source and preprocessing

Molecular data, including RNA‐seq expression, somatic mutations and Illumina 450 k DNA methylation array of LUAD and the corresponding clinical information, including AJCC‐tumour node metastasis (TNM) stages, gender, age, integrative stage classification, overall survival times and survival status were downloaded from the Cancer Genome Atlas (TCGA).^19^

### 2.2 Calculation of hypoxia score

Hypoxia score was calculated based on a 15‐gene expression signature (*ACOT7*, *ADM*, *ALDOA*, *CDKN3*, *ENO1*, *LDHA*, *MIF*, *MRPS17*, *NDRG1*, *P4HA1*, *PGAM1*, *SLC2A1*, *TPI1*, *TUBB6* and *VEGFA*)^20^ by gene set variation analysis (GSVA) algorithm^21^ as before.^22^ GSVA is a gene set enrichment (GSE) approach which assesses potential variation in pathway activity over a sample population in a nonparametric unsupervised manner.^21^ GSVA algorithm recieves the input of a gene expression matrix (log2 microarray expression values or RNA‐seq counts) and a database of gene sets and outputs sample‐wise GSE scores.^21^ In this study, we used ‘gsva’ function in R package ‘GSVA’, parameters were selected as default, and the Fragments per Kilobase Million (FPKM) expression profiles of LUAD and the above hypoxia 15‐gene expression signature were used as inputs to obtain GSVA scores for each sample. We considered the GSVA score of each LUAD sample as a hypoxia score, which represented the hypoxia status of each LUAD sample. Then the LUAD patients were classified into high‐hypoxia and low‐hypoxia groups according to the median score. The frequency distribution histogram of hypoxia score was drawn using R package ‘ggplot2’.

### 2.3 Clinical relevance investigation

Clinical parameters included pathologic T, pathologic N, pathologic M, age, gender, integrative stage classification and survival outcomes. The Wilcoxon rank‐sum test was used to assess the statistical differences in hypoxia score between groups in distant metastasis, lymph nodes, age and gender, while Kruskal–Wallis test was used when it comes to three or four groups such as tumour size and integrative stages. The Kaplan–Meier survival analysis was implemented to investigate the survival difference between high‐ and low hypoxia groups. *p* < 0.05 was considered statistically significant.

### 2.4 Identification of differentially expressed genes (DEGs)

‘DESeq2’ R package^23^ was utilized to identify DEGs between high‐hypoxia and low‐hypoxia groups. DEGs were screened with |log fold‐change (FC)| >1 and false discovery rate (FDR) corrected adjust *p*‐value <0.05. The R package ‘ggplot2’ was used to generate the volcano map to exhibit the distribution of DEGs.

### 2.5 GO and KEGG functional enrichment analysis

Gene ontology (GO) enrichment analysis, Kyoto Encyclopedia of Genes and Genomes (KEGG) pathway enrichment analysis and gene set enrichment analysis (GSEA) of the DEGs and differentially methylated probe (DMP)‐related genes were conducted using the ‘clusterProfiler’^24^ R package. *p*‐value <0.05 was considered statistically significant.

### 2.6 Analysis of LUAD mutation signature

Somatic mutation data were procured from the TCGA database. We chose the ‘Masked Somatic Mutation’ data in which the mutations had been processed by VarScan2.^25^ Somatic mutations included single‐nucleotide polymorphism (SNP), single‐nucleotide variant (SNV), deletion (DEL) and insertion (INS). ‘Maftools’^26^ R package was implemented to analyse and visualize Mutation Annotation Format (MAF) of LUAD hypoxia‐related somatic mutations based on the whole exome sequencing (WES) data. Besides, we used the function ‘mafCompare’ to perform Fisher test on 2 × 2 contingency table generated from two groups to find differentially mutated genes, and genes with *p*‐value <0.05 were considered as differentially mutated. The co‐occurrence and mutually exclusive mutations were authenticated using the Combinations of Mutually Exclusive Alterations (CoMEt) algorithm.^27^

### 2.7 Analysis of DNA methylation profiling data of LUAD

The R package ‘ChAMP’^28^ was utilised to analyse LUAD Illumina Infinium 450 k DNA methylation array data. First, we used the ‘champ.QC’ function to filter unnecessary CpGs. We then used the ‘champ. impute’ function to conduct imputation on missing values of beta matrix with ‘Combine’ parameter. Samples with NA value proportion above 20% and probes with NA value proportion above 20% will be removed, the left samples were further separated into the high‐hypoxia group (*N* = 213) and low‐hypoxia group (*N* = 237). Next, we applied the ‘champ. norm’ function with Beta MIxture Quantile dilation (BMIQ)^29^ to correct type‐II probe bias due to the reduced dynamic range. Finally, the differential methylation probes were obtained using champ. DMP function which applys 'limma' package ^30^ to identify. P values were adjusted by Benjamini–Hochberg (BH) method.

### 2.8 Identification of DNA methylation‐based molecular subtypes

Consensus clustering was conducted using the R package ‘ConsensusClusterPlus’^31^ to identify LUAD molecular subtypes based on the DMP sites. 80% of the samples were resampled to iterate 1000 times to obtain stable clustering results. Then, a Partitioning Around Medoids (PAM) algorithm with Pearson (1‐Pearson correlation) distance metric was used. The optimal clustering numbers were obtained according to the cumulative distribution function (CDF) plot and delta plot.

### 2.9 Constructing and evaluating the prognosis prediction model of LUAD

For the prognostic prediction Cox model with integrating signatures from multi‐omics data above, we only accepted LUAD samples with all signatures available. Then we began to construct the prognostic model. First, we used univariate Cox proportional hazards regression to screen the features with significant prognosis value using the ‘survival’ R package. With *p* < 0.05, a total of 953 features were obtained. Then we adopted LASSO regression to cut off some variables with less contribution using R package ‘glmnet’^32^ and retained 38 variables. Next, we utilized multivariate Cox regression with a stepwise procedure to acquire a prediction model containing 25 signatures. Risk scores for all LUAD samples were calculated by ‘predict’ function in the ‘survival’ R package.

To get the best combination of prognostic model variables, 420 samples were randomly classified into three identical parts, of which each part was alternately as the independent testing set, and another two parts were used as the training set. The ‘predict’ function in the R package ‘survival’ was used to predict the risk score of each LUAD sample according to the training model. Then, the LUAD patients were separated into a high‐risk group and a low‐ risk group based on their median risk score. In addition, the risk scores were used to reconstruct another Cox model with three clinical factors (gender, age and stage) to further evaluate the predictive effect. We performed the survival analyses based on risk sores and plotted receiver operating characteristic (ROC) curve for 1–5 years using the R package ‘time ROC’,^33^ and calculated the area under curve (AUC) value of the ROC curve and concordance index (C‐index) to access the predictive performance of the prognosis model.

## 3 RESULTS

### 3.1 Association analysis between hypoxia status and clinical factors in LUAD

**FIGURE. 1:**
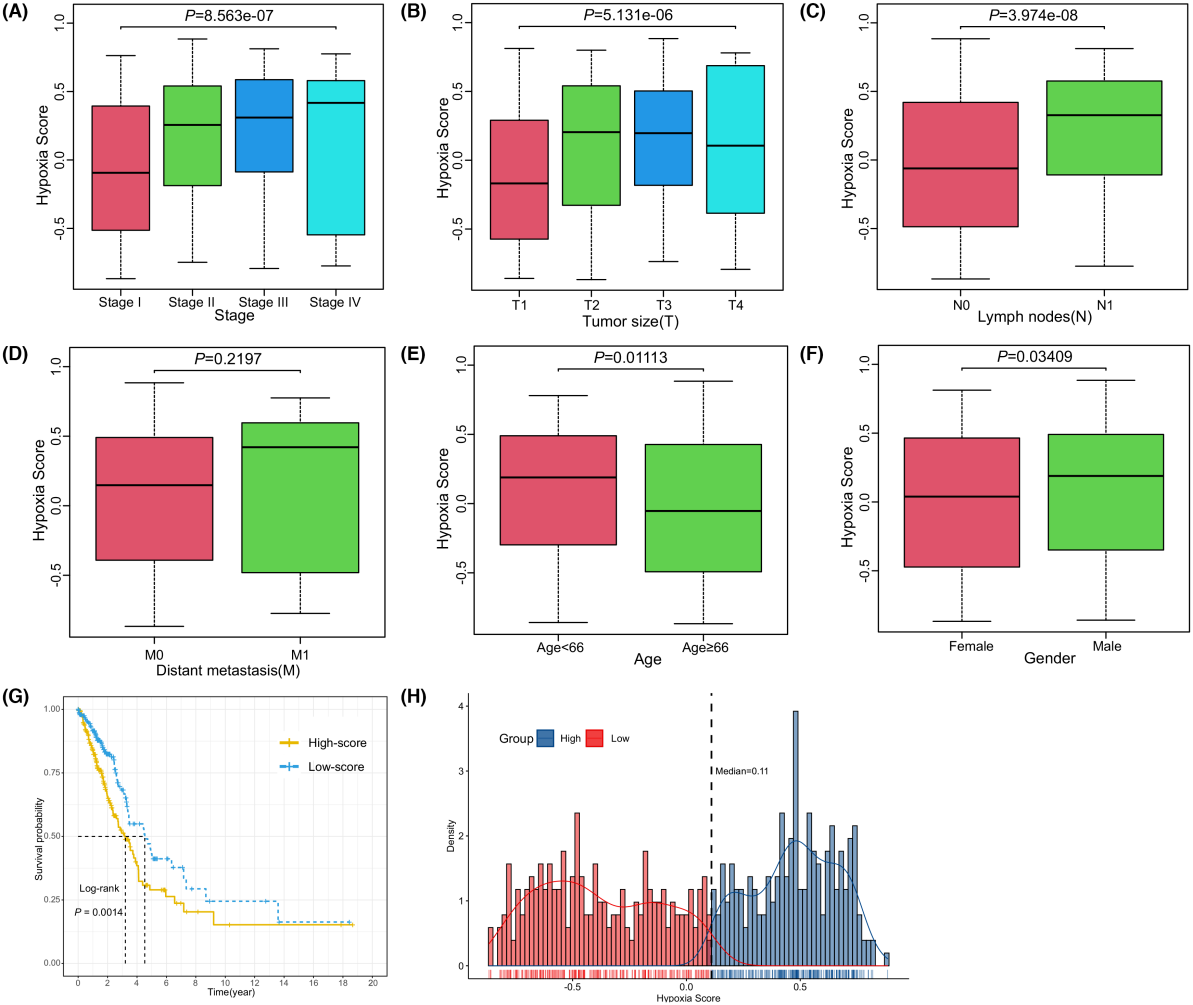
Relationship between hypoxia score and clinical features in LUAD. (A) TNM staging (I, II, III and IV). (B) Tumour size (T1, T2, T3 and T4). (C) Lymph nodes (N0 and N1). (D) Distant metastasis (M0 and M1). (E) Age (Age <66 and Age ≥66). (F) Gender (Female and Male). (G) Kaplan–Meier curves for overall survival between high‐ and low‐ hypoxia groups (log‐rank test, *p* = 0.0014). (H) Samples were split into low‐hypoxia and high‐hypoxia groups based on the median hypoxia value (*M* = 0.11). Red colour represents low‐hypoxia group, blue colour represents high‐hypoxia group. TNM, tumour node metastasis.

We computed the LUAD specimen's GSVA scores based on 15 hypoxia‐related genes, which were used as hypoxia scores to reveal the hypoxia status. The results showed that the hypoxia scores were distributed between −0.867 and 0.884 (Figure 1H). Five hundred and ten LUAD patients were classified into two groups based on the median value of hypoxia score (*M* = 0.108), which were named as high and low hypoxia groups respectively. The hypoxia scores differed significantly in TNM stage (*p* < 0.0001, Figure 1A), tumour size (*p* < 0.0001, Figure 1B), lymph nodes (*p* < 0.0001, Figure 1C), age (*p* = 0.01113, Figure 1E) and gender (*p* = 0.03409, Figure 1F). However, the difference in distant metastasis was not statistically significant (*p* = 0.2197, Figure 1D). Kaplan–Meier analysis showed that overall survival time significantly decreased with the increase of patients' hypoxia scores (*p* = 0.0014, Figure 1G).

### 3.2 Screening and functional enrichment analysis of hypoxia‐related genes

**FIGURE. 2:**
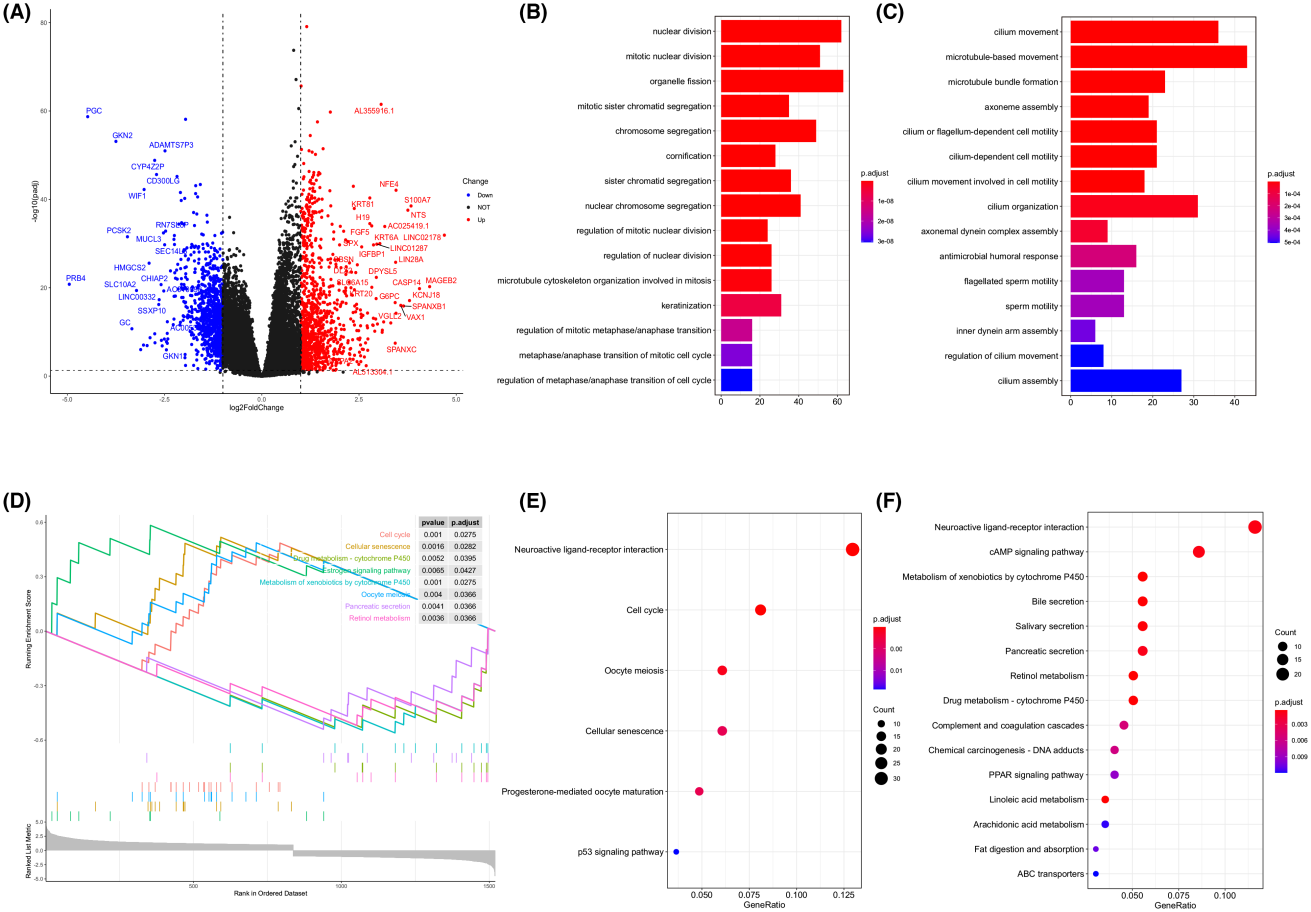
Identification and functional enrichment analysis of differentially expressed genes (DEGs) between low‐hypoxia and high‐hypoxia groups in lung adenocarcinoma (LUAD). (A) The volcano map shows differentially up‐regulated (red dots) and down‐regulated genes (blue dots). (B, C) Show the bar plots of GO functional items significantly enriched in differentially up‐regulated and down‐regulated genes, respectively. (D) Gene set enrichment analysis (GSEA) shows the significantly enriched biological processes. (E, F) Show the bar plots of Kyoto Encyclopedia of Genes and Genomes (KEGG) pathways significantly enriched in differentially up‐regulated and down‐regulated genes, respectively.

Differentially gene expression analysis was conducted between the two hypoxic groups. A total of 1820 DEGs, which were considered as hypoxia‐related genes, were screened, including 955 up‐regulated genes and 865 down‐regulated genes (Figure 2A). The top 15 GO terms of up‐regulated genes implied that these genes were mainly enriched in biological processes related to cell division and proliferation, demonstrating that these genes may produce an indispensable role in the process of tumour growth and progression (Figure 2B). Correspondingly, down‐regulated genes were primarily involved in cilium‐related biological processes (Figure 2C), which has been shown to be associated with cancer previously.^34^ Interestingly, KEGG pathway enrichment analysis revealed that both up‐regulated DEGs and down‐regulated DEGs were mainly associated with neuroactive ligand‐receptor interaction (Figure 2F). Ligand‐receptor interactions between malignant tumour cells and other non‐tumour cells in the TME play a vital role in promoting tumour progression.^35^ GSEA analysis displayed that the up‐regulated pathways included cell cycle and cellular senescence while drug metabolism‐cytochrome P450 and metabolism of xenobiotics by cytochrome P450 pathways were down‐regulated (Figure 2D).

### 3.3 Comparison of somatic mutations under different hypoxia levels

**FIGURE. 3:**
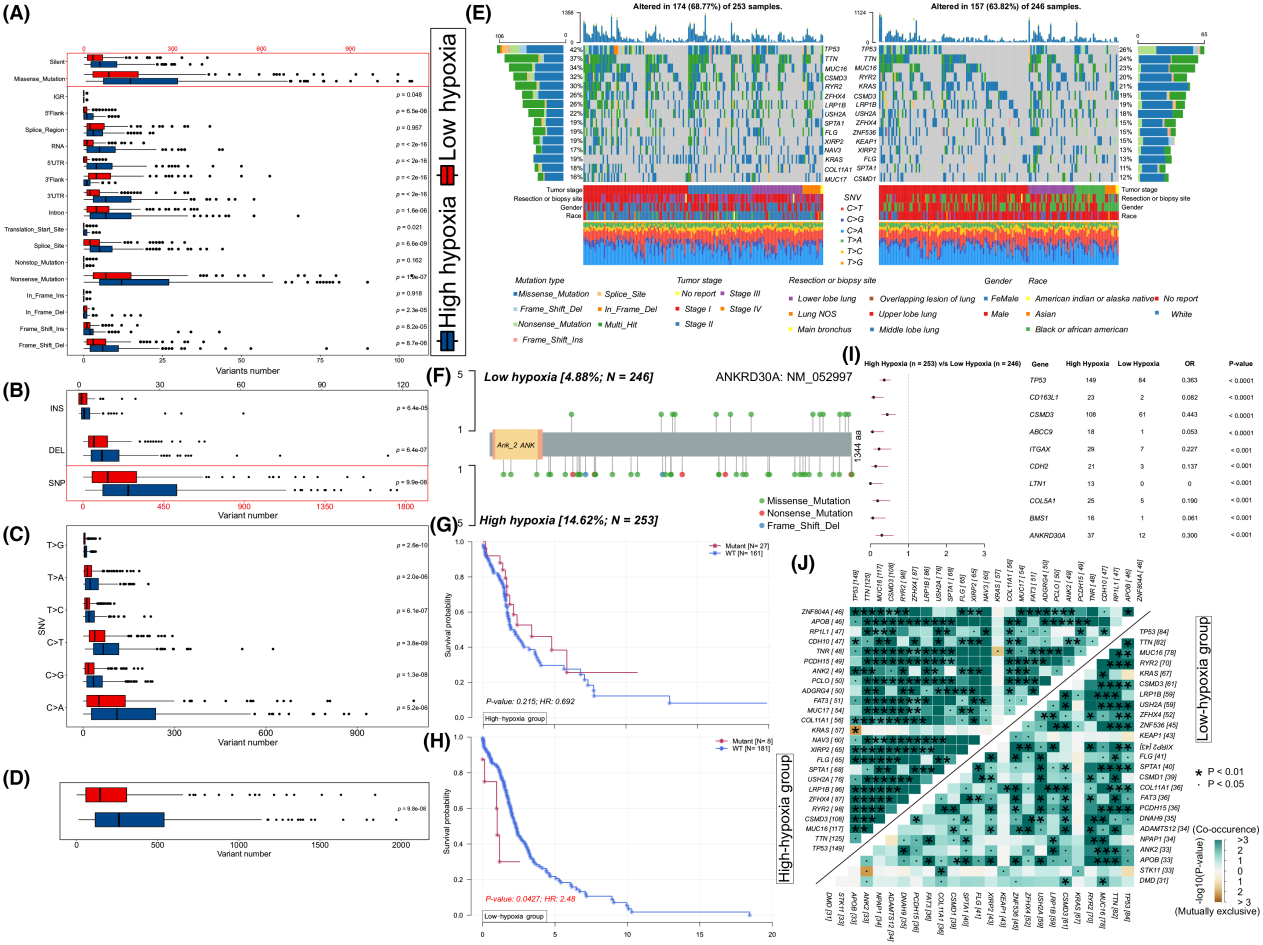
Somatic mutation profiling between low‐hypoxia and high‐hypoxia groups. (A–D) The box plots show the contrasts of variant numbers of (A) effects classification for every variant type, (B) SNV, (C) DEL, INS and SNP, and (D) total mutations between low‐hypoxia group and high‐hypoxia groups. (E) Oncoplots show the top 15 genes with the highest mutation frequency in the high‐hypoxia group (left side) and low‐hypoxia group (right side). The upper panel shows the TMB of each lung adenocarcinoma (LUAD) samples. The central panel shows the mutation types of each LUAD samples. The lower panel shows each LUAD sample's clinical features (tumour stage, resection or biopsy site, gender and race) and SNV types. Each column represents one LUAD patient. The left and the right panel of bar plots show the frequency of mutated genes and corresponding mutation type in the high‐hypoxia group and low‐hypoxia group, respectively. The bottom part shows the legend of clinical features and variants types. (F) The lollipop diagram reveals the mutation site information on the *ANKRD30A* gene. (G, H) Kaplan–Meier curves illustrate that *ANKRD30A* mutation is an independent factor of overall survival in high‐hypoxia (G) and low‐hypoxia groups (H). (I) Forest plot shows the top 10 differentially mutated genes with the most significance between high‐hypoxia and low‐hypoxia groups. (J) The heatmap demonstrates the 25 top mutually exclusive and co‐occurring gene pairs. Green colour represents co‐occurring tendency, brown colour represents exclusive tendency. The symbol represents the statistical significance for each gene pairs.

Missense mutations accounted for the majority (~60%) of somatic mutation in both groups with high hypoxia scores and low hypoxia scores (Figure 3A). Therefore, we further analysed the other types of somatic mutations and revealed their potential effects on LUAD. On the whole, the number of sample variants in group with high hypoxia scores (*M* = 261.0) was obviously greater than that in group with low hypoxia scores (*M* = 145.0) (Figure 3D). A total of 93,958 and 57,373 SNVs were detected across all samples in both the group with high hypoxia scores and group with low hypoxia scores, respectively. Cytosine to adenine (C > A) was the most usual type of base substitution in both groups. The number of mutations in all six types of SNV was remarkably higher in the group with high hypoxia scores than in the group with low hypoxia scores (Figure 3C). Meanwhile, the number of SNPs, DELs and INSs in the group with high hypoxia scores was also outdistanced than that in the group with low hypoxia scores (Figure 3B). Moreover, the ratios of transversion (Tv) to transition (Ti) were approximately 2:1 across all SNVs and held steady in these two groups (Figure S1). However, the difference in the proportion of each mutation type was not statistically significant (Figure S1).

Meanwhile, there were 167 frequently mutated genes (mutated in more than 10% of the samples) in the group with high hypoxia scores and 51 in the group with low hypoxia scores, respectively. The waterfall diagrams revealed that the mutation rates of three genes (*TP53*, *TTN* and *MUC16*) were the highest in two groups (Figure 3E). Subsequently, co‐occurring and exclusive mutations analysis showed that, in contrast to the generalized co‐occurrence circumstance, three distinct relationships were exhibiting mutually exclusive mutations in two groups (*KRAS*‐*TP53*, *KRAS*‐*TNR* and *STK11*‐*ANK2*; Figure 3J). As reported, co‐occurring mutations of *TP53* and *STK11* in *KRAS*‐positive LUAD cohort shown a poorer survival,^36^ suggesting they may be important in LUAD. In addition, a total of 497 differentially mutated genes were detected (Figure 3I). Furthermore, a lollipop plot showed that there are different mutation sites of *ANKRD30A* between the two hypoxic groups (Figure 3F). And in the group with low hypoxia scores, LUAD patients with *ANKRD30A* mutation had significantly lower survival and worse prognosis than those with wild‐type, but a similar situation was not observed in the group with high hypoxia scores (Figure 3G,H).

### 3.4 Distinct DNA methylation prognosis subgroups in LUAD

**FIGURE. 4:**
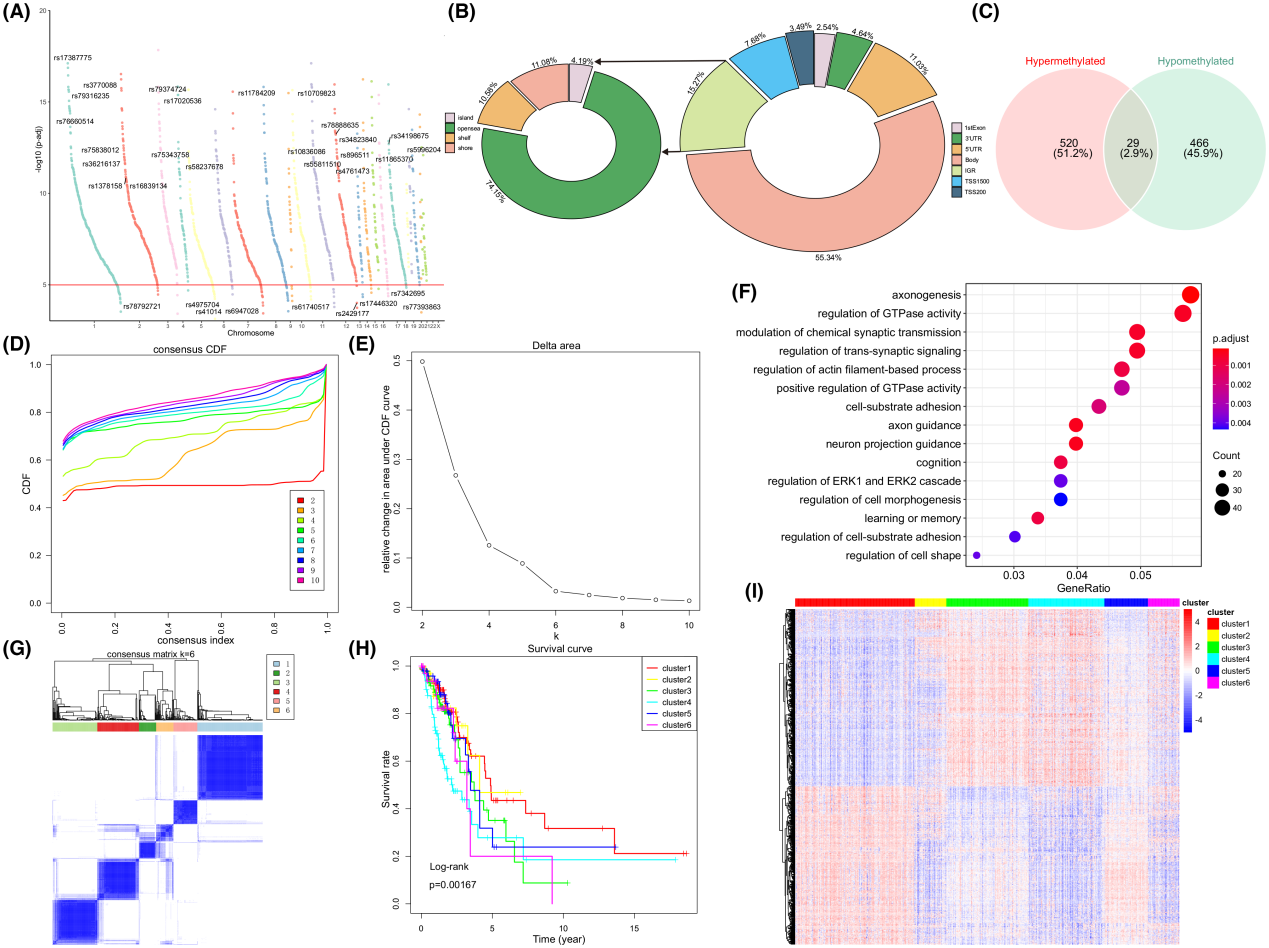
Identification of DNA methylation features in lung adenocarcinoma (LUAD). (A) Manhattan plot of DNA methylation differences in high‐hypoxia and low‐hypoxia groups. (B) Distribution of differentially methylated probes (DMP) in genomic region (left side) and genomic features (right side). (C) Venn diagram shows 29 gene overlapped between hypermethylation and hypomethylation group. (D) Cumulative distribution function (CDF) curve for each category number *k* = 2–10. (E) CDF delta area curve (*k* = 2–10), the optimal *k* = 6. (F) The GO terms of top 15 enriched biological processes for DMP‐related genes. (G) Heatmap of the corresponding consensus matrix when *k* = 6. (H) Kaplan–Meier survival curves for 6 DNA methylation clusters (log‐rank, *p* = 0.00167). (I) The heatmap corresponding to six clusters.

A total of 2004 DMPs (1698 genes) were finally identified (deltaBeta ≥0.1, FDR *p*‐value <0.05, Figure 4A). Among them, 832 probes (41.5%) involving 549 genes were significantly hypermethylated, and 1172 probes (58.5%) involving 495 genes were significantly hypomethylated in the high‐hypoxia group, among which 29 genes were the same (Figure 4C). Most of DMPs were located in the gene body (55.34%) and noncoding intergenic region (IGR, 15.27%), in the meantime, DMPs had the highest frequency of distribution in the open sea (74.15%) (Figure 4B).

Meanwhile, GO function enrichment analysis revealed DMP‐related genes' potential roles were tumour‐related neurobiological processes and cell signal transduction (Figure 4F), suggesting that hypoxia may be related to abnormal methylation, and may affect the tumour progression through the recognition and participation of neural pathways.

Consensus clustering of the 1015 DMPs was used to identify potential prognostic molecular subgroups of LUAD with distinct DNA methylation. We found that CDF began to plateau when *k* = 6 (Figure 4D,E). We also used the consistency matrix to confirm the optimal cluster numbers (Figure 4G) and thus a well‐differentiated six clusters were revealed (Figure 4I). In addition, the survival curves of different clusters showed obviously differences in overall survival among these clusters, suggesting that DNA methylation patterns may affect the prognosis of patients with LUAD (Figure 4H).

### 3.5 Construction of LUAD prognostic prediction model with multiple‐omics data

**FIGURE. 5:**
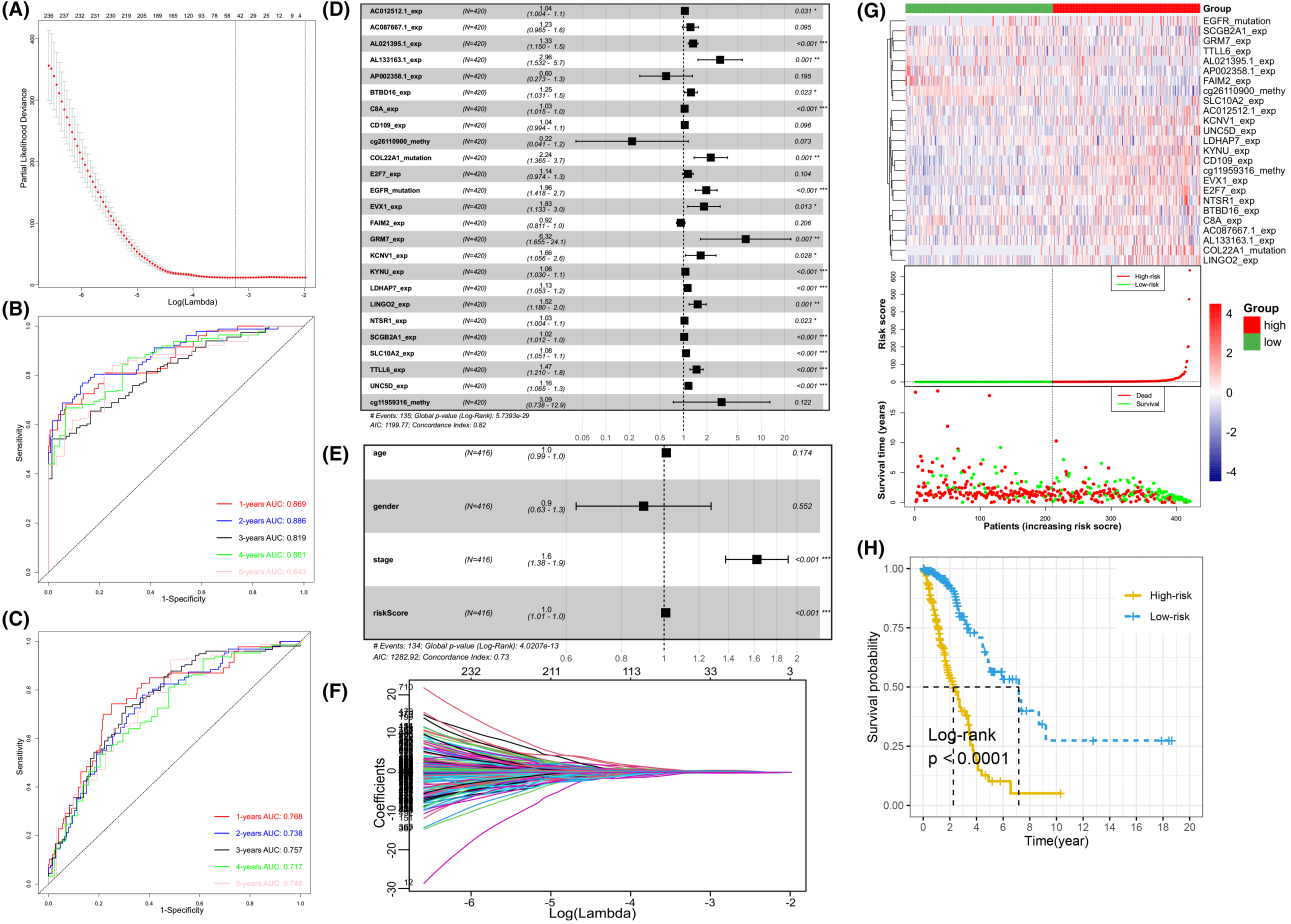
Construction of a predictive model. (A) Selection of the optimal parameter (λ) in LASSO coefficient profiles, and the optimal log (λ) = −3.2. (B) Time‐dependent ROC curves analysis of the risk scores for predicting 1–5 years overall survival rates. (C) Time‐dependent ROC curves analysis of the risk scores and clinical characteristics for predicting 1–5 years overall survival rates. (D) Forest plot of the relation between the overall Cox model with 25 features and overall survival time. (E) Forest plot of the relation between risk scores combined with clinical characteristics and overall survival time. (F) LASSO coefficient profiles. (G) The scatter plots represent the distribution of the risk scores and survival statuses of high‐risk and low‐risk lung adenocarcinoma (LUAD) samples, respectively. The heatmap shows the expression profile distribution of 25 features. (H) Kaplan–Meier survival curves indicate that risk score was an independent risk factor for overall survival in LUAD patients.

Hypoxia plays an irreplaceable role in multiple‐omics dimensions of LUAD from the results of above sections. Therefore, it is reasonable to believe that hypoxia is also closely correlated to patients' survival prognosis with LUAD. First, we performed univariate Cox regression analysis to screen all multi‐omics features and a total of 953 features were identified, consisting of 132 DEGs, 325 mutations and 496 DMPs. Then, we further removed variables with fewer contributions through LASSO regression analysis. With the most suitable parameter log (λ) = −3.2 (Figure 5A,F), 36 features were retained for constructing a multivariate Cox regression model. Due to the absence of matching suitable other source's multi‐omics data, we stochastically split TCGA‐LUAD specimens into training and test sets. Each 1/3 of the specimens (*N* = 140) rotated as the independent test set, and the remaining 2/3 (*N* = 280) was used as the corresponding independent training set. Therefore, three models were constructed. As results, the average concordance index (C‐index) of these training models was 0.822. The average AUCs of the ROC curves of the training set correspond to 0.871, 0.814 and 0.846 at 1‐year, 3‐year and 5‐year survival, respectively (Table 1; Figure S5). As for the test sets, the predictive performance decreased slightly, with the mean AUC values of 0.809, 0.790 and 0.809 corresponding to 1‐year, 3‐year and 5‐year survival. Then, we bonded all TCGA LUAD samples together and produced an overall Cox model incorporating with 25 features (Figure 5D). The contributions of these features on the holistic model are shown in nomogram (Figure S2). The total score in the nomogram is inversely interrelated with patient outcome, that is, the higher the score, the worse the patient outcome. In short, the gene expression levels of *AC012512.1*, *AL021395.1*, *AL133163.1*, *BTBD16*, *C8A*, *EVX1*, *GRM7*, *KCNV1*, *KYNU*, *LDHAP7*, *LINGO2*, *NTSR1*, *SCGB2A1*, *SLC10A2*, *TTLL6* and *UNC5D*, the mutations of *COL22A1* and *EGFR* were significantly associated with worse prognosis. Correspondingly, the expression of *AP002358.1* and *FAIM2*, the methylation level of DMP *cg26110900* probe had the opposite effects. The final Cox model has good prediction power with AUC values of survival rates at 1‐year, 3‐year and 5‐year corresponding to 0.863, 0.826 and 0.846, respectively (Figure 5B). On the other hand, all LUAD specimens' risk scores were computed by the above model and were differentiated as group with high‐risk and low‐risk according to their median risk value. The risk of death in patients with LUAD showed a consistent trend with the risk score, while the survival time had an opposite trend with the risk score (Figure 5G). The survival curve indicated that the overall survival of LUAD samples in group with higher risk score was worse than that of LUAD samples in group with lower risk score (*p* < 0.001, Figure 5H).

**TABLE 1:** The concordance indexes, 1‐, 3‐, and 5‐year AUC values and mean values of trained models.

Index	Model 1		Model 2		Model 3		Average	
	Training set 1	Test set 1	Training set 2	Test set 2	Training set 3	Test set 3	Training set	Test set
1‐year AUC	0.869	0.873	0.880	0.776	0.864	0.779	0.871	0.809
3‐year AUC	0.810	0.821	0.840	0.758	0.793	0.790	0.814	0.790
5‐year AUC	0.838	0.893	0.891	0.658	0.809	0.875	0.846	0.809
C‐index	0.815		0.826		0.824		0.822	

Abbreviations: AUC, area under curve; C‐index, concordance index.

After, we followed the same approach as above for the individual omics data to ascertain whether the combined effects of these three different omics types indeed outperformed the separate effects. The results showed that compared to the combined model, no single type of feature could provide sufficiently powerful prognostic prediction (Figure S3). In addition, when constructing the model with differentially mutated genes instead of frequently mutated genes, we obtained a predictive model containing 22 variables (Figure S4), but the performance did not improve. Furthermore, given the possible predictive values of important clinical factors for overall survival time, we combined the risk scores in the final model with several clinical factors (gender, age, stage) to construct another prognostic model. Regrettably, the results gave us hints that the prediction ability of the new Cox model including clinical factors did not improve, with C‐index of 0.732 (Figure 5E) and AUC values of 1‐year, 3‐years and 5‐years were equal to 0.768, 0.757 and 0.748, respectively (Figure 5C). Therefore, we only retained the multivariate Cox prognostic model embodying the above 25 multi‐omics features because of its best prognostic prediction power.

## 4 DISCUSSION

Targeting therapy has transformed the treatment strategy of patients with LUAD and improved survival rate, but only a few selected patients have benefit.^37^ Drug resistance of tumour cell involves multiple mechanisms, with hypoxia being one of the pivotal factors affecting the cell expression programs and resulting in treatment‐resistant^15^ and radiotherapy resistance.^38^ Hypoxia is a complicated phenomenon involving hundreds of molecules.^39^ Besides, genes do not function in isolation.^40^ Therefore, prognosis models with integrating multiple hypoxia‐related genes could help increase the precision of prognosis prediction.^41^ Until now, several hypoxia‐related gene features have been built to predict survival rate of certain tumour types, such as ovarian cancer,^42^ renal clear cell carcinoma,^43^ hepatocellular carcinoma,^44^ breast cancer,^45^ melanoma^46^ and prostate cancer.^47^ Although the role of hypoxia‐related genes in prognosis prediction of LUAD has received extensive attention in recent years,^48^, ^49^, ^50^, ^51^ the role of hypoxia in LUAD was not fully elucidated. Several studies have combined hypoxia and immunization to predict the prognosis of patients with LUAD, and the constructed models also have good predictive performances.^51^, ^52^ Therefore, it is still necessary to construct more accurate prediction models of LUAD from multi‐omics levels. Considering that tumour hypoxia is a complex biological process which involves complicated regulation networks and multiple signal pathways among beaucoup genes. In addition to genetic abnormalities, LUAD is also controlled by epigenetic mechanisms such as DNA methylation. We constructed a prognostic model from three omics perspectives of gene expression, somatic mutation and DNA methylation, which could reflect the relationship between hypoxia and prognosis in LUAD patients more precisely.

In this study, we computed LUAD patients' hypoxia scores with GSVA algorithm based on hypoxia‐related genes, and divided the patients into group with high hypoxia scores and group with low hypoxia scores according to the median score. Association analysis showed that there were statistically significant correlations between hypoxia score and age, sex, stage, pathological T and pathological N (Figure 1).

GO analysis indicated that up‐regulated genes mainly played roles in nuclear division, organelle fission and chromosome segregation (Figure 2B), indicating that they may promote tumorigenesis and tumour cell proliferation. While, the biological processes concerned with cilia, such as cilium movement and microtubule‐based movement (Figure 2C), appeared to have unique roles in down‐regulated genes. The development of tumours was often accompanied by the loss of primary cilia.^53^, ^54^ Interestingly, KEGG pathway analysis demonstrated that neuroactive ligand‐receptor interaction signalling pathway was significantly enriched in both up‐ and down‐regulated DEGs (Figure 2E,F). Ligand‐receptor interactions between malignant tumour cells and other non‐tumour cells in the TME played a vital role in promoting tumour progression.^35^ The top 15 GO terms and KEGG pathways did not show hypoxia‐related processes, however, some hypoxia‐related GO biological process terms were indeed statistically significant such as response to hypoxia, response to decreased oxygen levels and cellular response to decreased oxygen levels (*p* < 0.05). In addition, HIF‐1 signalling pathway in KEGG was also significant (*p* < 0.05). We speculated that this may be caused by multiple functions and widespread impact of hypoxia‐related genes on other genes in cancer.

Furthermore, the results of GSEA analysis on DEGs manifested that the pathways associated with cell cycle, cellular senescence and the oestrogen signalling pathway were up‐regulated, whereas the genes related to the drug metabolism‐cytochrome P450, metabolism of xenobiotics by cytochrome P450 and retinol metabolism pathways were down‐regulated (Figure 2D). Previous studies have confirmed that, hypoxia affects LUAD from multiple cancer pathways, including cell cycle,^55^ cell division,^56^ oestrogen signalling pathway,^57^, ^58^ cytochrome P450^59^ and cellular senescence.^60^

Somatic mutation analysis gave us hints that the LUAD samples in the group with higher hypoxia scores were more prone to TP53 mutation (Figure 3E), a well‐known frequently mutated gene in LUAD.^61^, ^62^ Consistent with previous studies,^63^ TP53 mutation patients survive for a shorter period, and HIF‐1/mutant p53 crosstalk, mediated by hypoxia, is an innovative potential therapeutic target for LUAD.^64^ Furthermore, three distinct relationships were exhibiting mutually exclusive mutations in the two groups (*KRAS*‐*TP53*, *KRAS*‐*TNR* and *STK11*‐*ANK2*) according to co‐occurring and exclusive mutations analysis (Figure 3J). Recent study supports our findings too, co‐occurring mutations of *TP53* and *STK11* in *KRAS*‐positive LUAD cohort was associated with poor survival.^36^ Meanwhile, our study showed a statistical discrepancy in survival time between the *ANKRD30A* mutation and the wild‐type (Figure 3G,H), suggesting that the expression of *ANKRD30A* may relate to the prognosis of LUAD patients, and hypoxia may also be associated with the expression of *ANKRD30A*.

Hypoxia affects DNA methylation through HIF‐dependent mechanism, which regulates the expression of hypoxia‐responsive gene in tumour cells.^65^ In this study, DMPs maybe concerned with axonogenesis and axon guidance (Figure 4F), and therefore involved in cell migration and tumour metastasis, and further contribute to LUAD progression. Several studies have supported the above findings too.^66^, ^67^, ^68^ Besides, most DMPs were localized to the gene body and noncoding intergenic region, and the distribution frequency of DMPs is the highest in the open sea (Figure 4B). LUAD is highly heterogeneous at multiple levels, comprising clinical, cellular and molecular aspects.^69^, ^70^, ^71^, ^72^ In our study, consistent cluster analysis identified 6 LUAD subgroups with different potential prognosis (Figure 4G). The survival curves of 6 clusters suggested obvious differences in overall survival among each cluster (Figure 4H).

Subsequently, we established a prognosis Cox model with 25 features (Figure 5D), including *AC012512.1*, *AC087667.1*, *AL021395.1*, *AL133163.1*, *AP002358.1*, *BTBD16*, *C8A*, *CD109*, *cg26110900*, *COL22A1*, *E2F7*, *EGFR*, *EVX1*, *FAIM2*, *GRM7*, *KCNV1*, *KYNU*, *LDHAP7*, *LINGO2*, *NTSR1*, *SCGB2A1*, *SLC10A2*, *TTLL6*, *UNC5D* and *cg11959316*. Some of these genes have been shown to be induced by hypoxia. For example, in hepatocellular carcinoma cell lines, hypoxia could induce *E2F7* expression and promote sirolimus resistance. And *E2F7* could activate downstream genes by stabilizing HIF‐1α.^73^ In breast cancer cell lines, hypoxia can modulate *EGFR* expression and downstream signalling in a DNA methylation‐specific and HIF‐dependent manner, altering the response to anti‐*EGFR* therapy.^74^ In the liver metastatic tissue derived from patients of colorectal cancer, *SCGB2A1* was identified as a novel hypoxia‐inducible gene and prognostic marker associated with chemoresistance and radioresistance.^75^ In hypoxia condition, overexpression of *TTLL6* significantly lowered the IC50 of cisplatin (CDDP) and increased the CDDP‐induced apoptosis in EC109/ CDDP cells.^76^ Moreover, hypoxia downregulates *LPP3* (cg11959316) during cancer cell invasion.^77^ According to our literature search results, the remaining genes have not yet been found to be directly related to hypoxia. Further researches that focuses on these potential prognostic‐related genes should be performed in future and may find new biomarkers of LUAD. Meanwhile, these genes may lead to gene‐mediated molecular targeting therapy. In addition, some of these genes have been proved to be related to lung cancer. For instance, *CD109* regulates LUAD invasion through TGF‐β signalling pathway, and up‐regulation of *CD109* promotes the epithelial‐to‐mesenchymal transition and stemness properties of LUAD by activating the Hippo‐YAP signalling pathway.^78^, ^79^ *E2F7*‐*RAD54L* axis facilitates the LUAD progress through the mTORC1 signalling pathway.^80^ *EGFR* mutation was one of the most common somatic mutations in LUAD,^59^ present in about 20%^81^ and strongly affected LUAD patients' prognosis.^82^ Moreover, *FAIM2* could boost NSCLC cell growth and bone metastasis via activation of the Wnt/β‐Catenin pathway.^83^ Meanwhile, *NTSR1* was over‐expressed in LUAD^84^ and proved to be linked to the lung cancer's progression.^85^ In the next place, in NSCLC cells, the over‐expression of *SLC10A2* could further inhibit cell proliferation and migration and accelerate cell apoptosis under the treatment of Bexarotene.^86^ Furthermore, *LPP3* (*cg11959316*) expression was consistently down‐regulated in lung cancer patients,^87^ and there was a relationship between lower *LPP3* expression and worse overall survival in LUAD patients.^88^ However, there was no overlap between these genes in the prognostic model and the 15 gene expression signatures used for hypoxia score calculation. We suspected that these genes may interact with each other and influence the prognosis of LUAD patients by participating in specific signalling pathways.

The Cox model has good performance with high C‐index and AUC values of ROC (Figure 5B). Then, LUAD patients were splited into group with high risk and low risk on the basis of the median value‐at‐risk (Figure 5G). At the same time, the risk score can independently predict the prognosis of patients with LUAD. In the end, to predict the survival probability of LUAD patients, a nomogram was also drawn, with good predictive performance (Figure S2).

Our study might provide some useful information for hypoxia‐targeted personalized treatment of LUAD. However, there were also some deficiencies in our research. First, our data source was TCGA database only, the reliability of the constructed prediction model applied to data from other sources remains to be further studied. Second, the prognostic genes screened need further experiments to verify their relationship with LUAD under hypoxia conditions.

## 5 CONCLUSION

A Cox model was established based on 25 hypoxia‐related features, including *AC012512.1*, *AC087667.1*, *AL021395.1*, *AL133163.1*, *AP002358.1*, *BTBD16*, *C8A*, *CD109*, *cg26110900*, *COL22A1*, *E2F7*, *EGFR*, *EVX1*, *FAIM2*, *GRM7*, *KCNV1*, *KYNU*, *LDHAP7*, *LINGO2*, *NTSR1*, *SCGB2A1*, *SLC10A2*, *TTLL6*, *UNC5D* and *cg11959316*. The frequencies of *TP53* mutation were dramatically higher and the survival time was shortened in LUAD patients with higher hypoxia scores. Additionally, the hypoxia scores differed significantly in TNM stage, tumour size, lymph nodes, age and gender. This study could expand the perspective of hypoxia‐related genes research and prognosis estimation of LUAD.

## AUTHOR CONTRIBUTIONS

**Qineng Luo:** Data curation (lead); formal analysis (lead); investigation (lead); methodology (lead). **Xing Li:** Formal analysis (supporting). **Zixing Meng:** Formal analysis (supporting). **Hao Rong:** Formal analysis (supporting). **Yanguo Li:** Formal analysis (supporting); investigation (supporting); methodology (supporting). **Guofang Zhao:** Investigation (supporting). **Huangkai Zhu:** Investigation (supporting). **Lvjun Cen:** Investigation (supporting). **Qi Liao:** Data curation (lead); formal analysis (lead); funding acquisition (lead); investigation (lead); project administration (lead).

## FUNDING INFORMATION

This work was supported by the Zhejiang Provincial Natural Science Foundation of China (Grant No: LY21C060002); the National Natural Science Foundation of China (Grant No: 31970630); the Ningbo Natural Science Foundation of China (Grant No: 2021J124), the Fundamental Research Funds for the Provincial Universities of Zhejiang (No: SJLZ2021001); the Zhejiang Medicine and Health Science and Technology Project (Grant No: 2022KY1138), the Ningbo Health Branding Subject Fund (Grant No: PPXK2018‐05).

## CONFLICT OF INTEREST STATEMENT

The authors declare no conflicts of interest.

## Supporting information


Figure S1.

Figure S2.

Figure S3.

Figure S4.

Figure S5.

## Data Availability

The raw data of this study are derived from the TCGA database (https://portal.gdc.cancer.gov/), which is publicly available database.
