# Supplementary figures and images for: Identification of hypoxia‐related gene signatures based on multi‐omics analysis in lung adenocarcinoma
Source: J Cell Mol Med. 2023 Nov 27;28(2):e18032. doi: 10.1111/jcmm.18032 (PMC10826438; doi:10.1111/jcmm.18032)

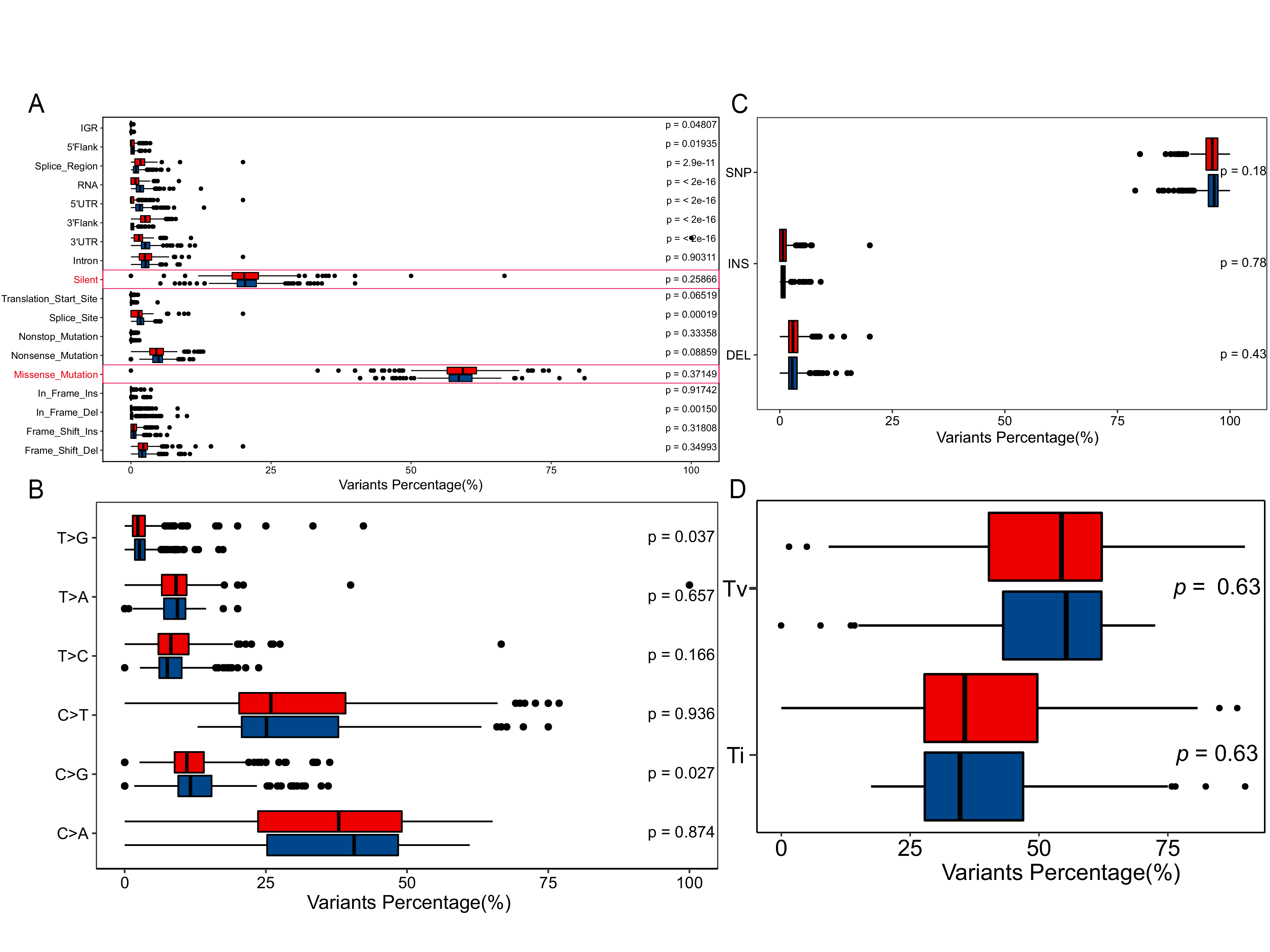

Supplement: Supplementary file 1 — Figure S1. [file JCMM-28-e18032-s003.tif]

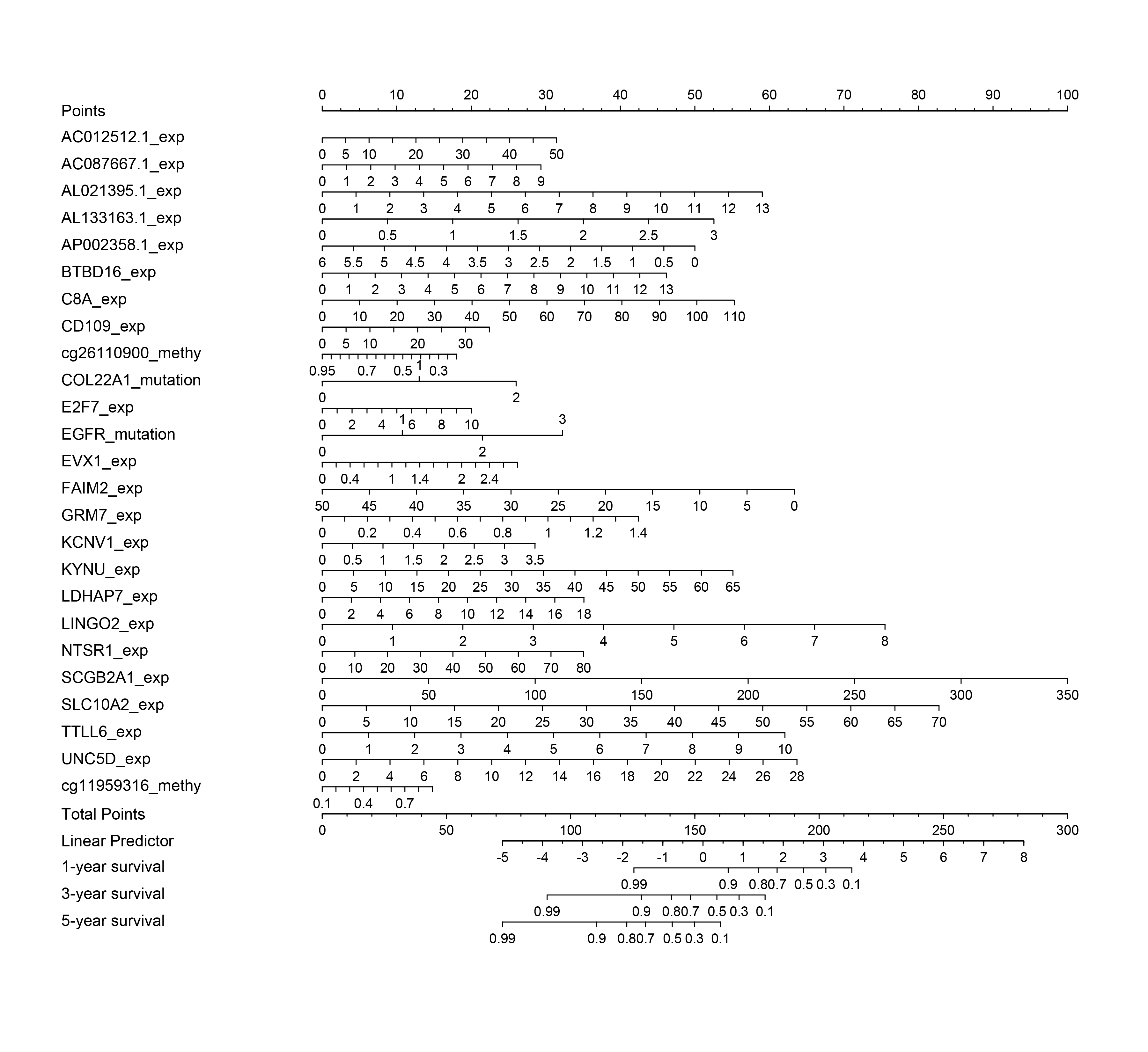

Supplement: Supplementary file 2 — Figure S2. [file JCMM-28-e18032-s005.tif]

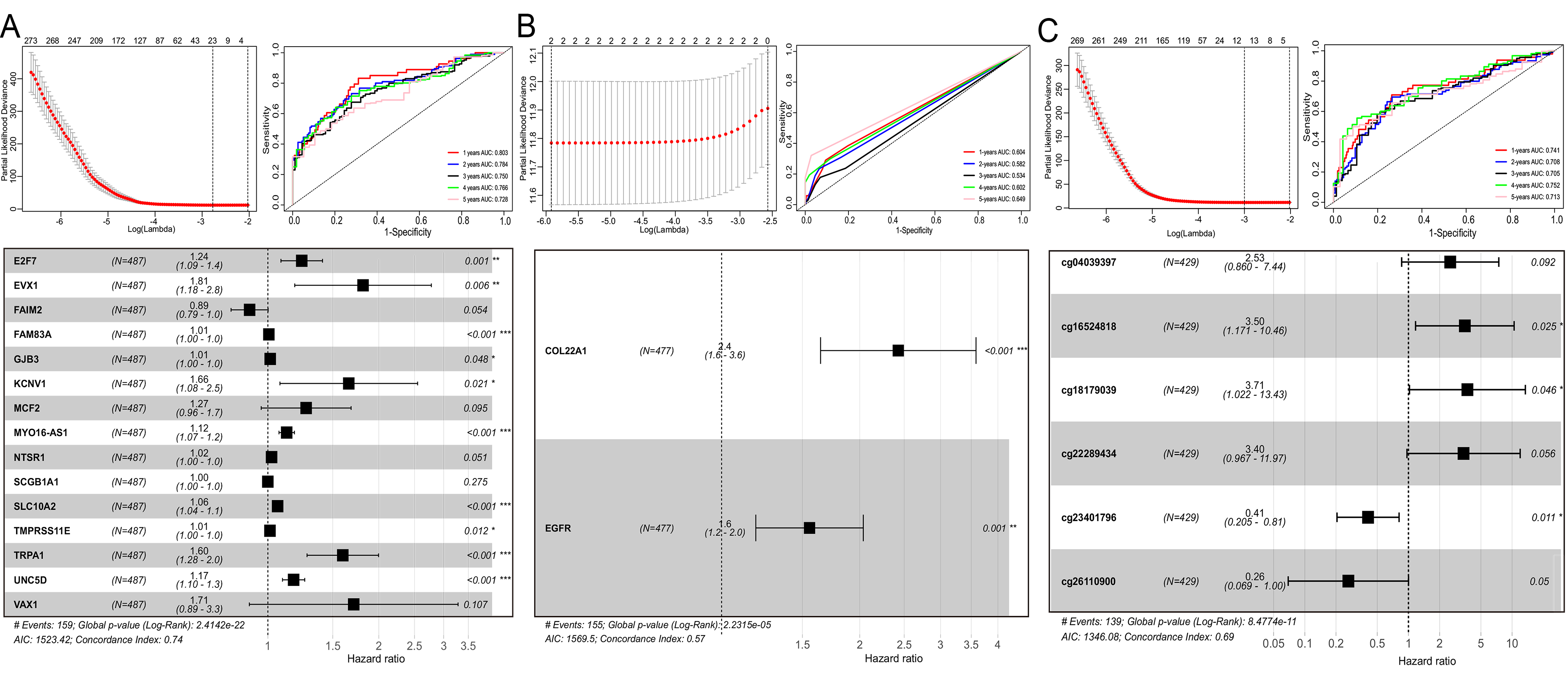

Supplement: Supplementary file 3 — Figure S3. [file JCMM-28-e18032-s001.tif]

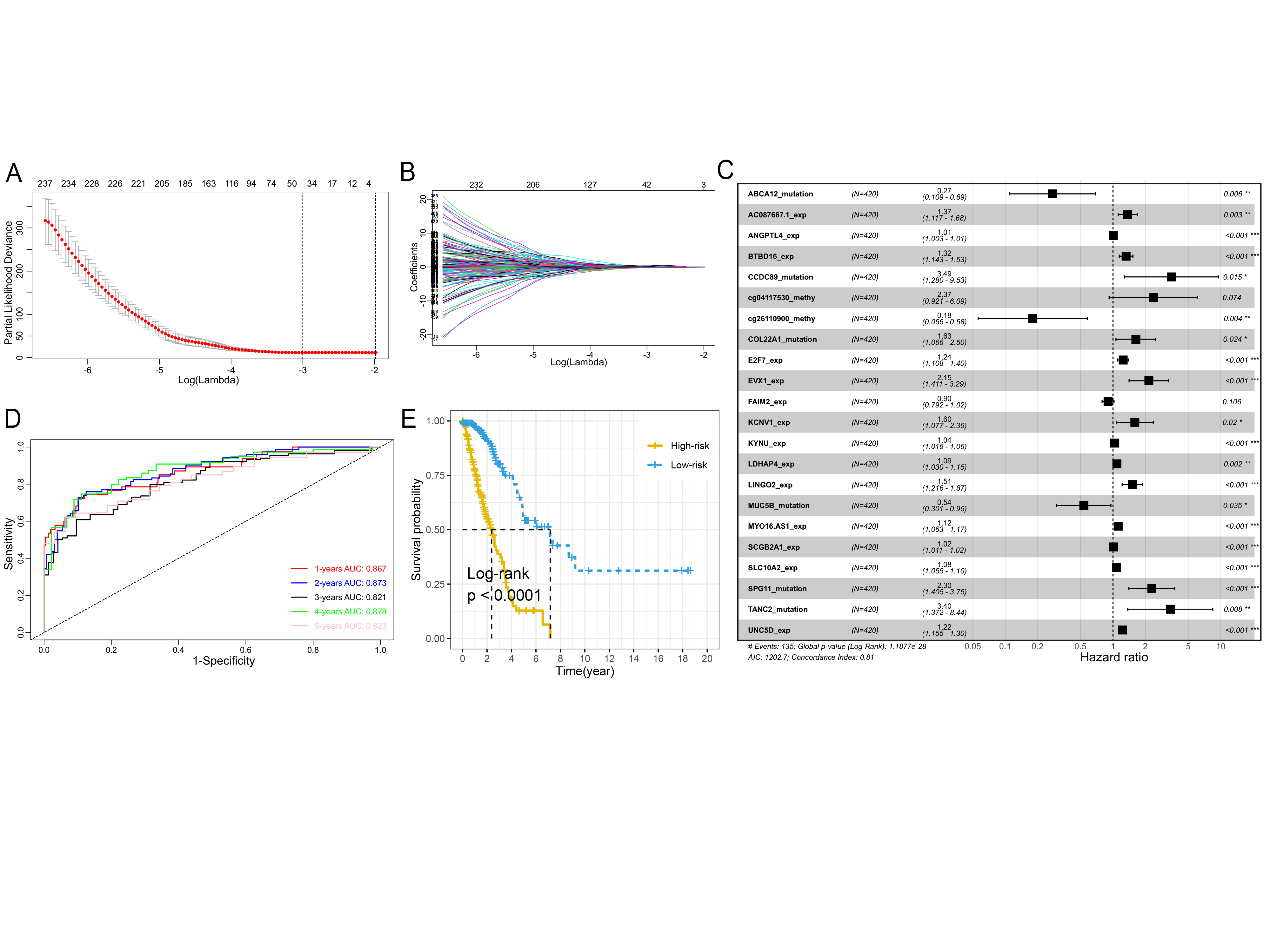

Supplement: Supplementary file 4 — Figure S4. [file JCMM-28-e18032-s002.tif]

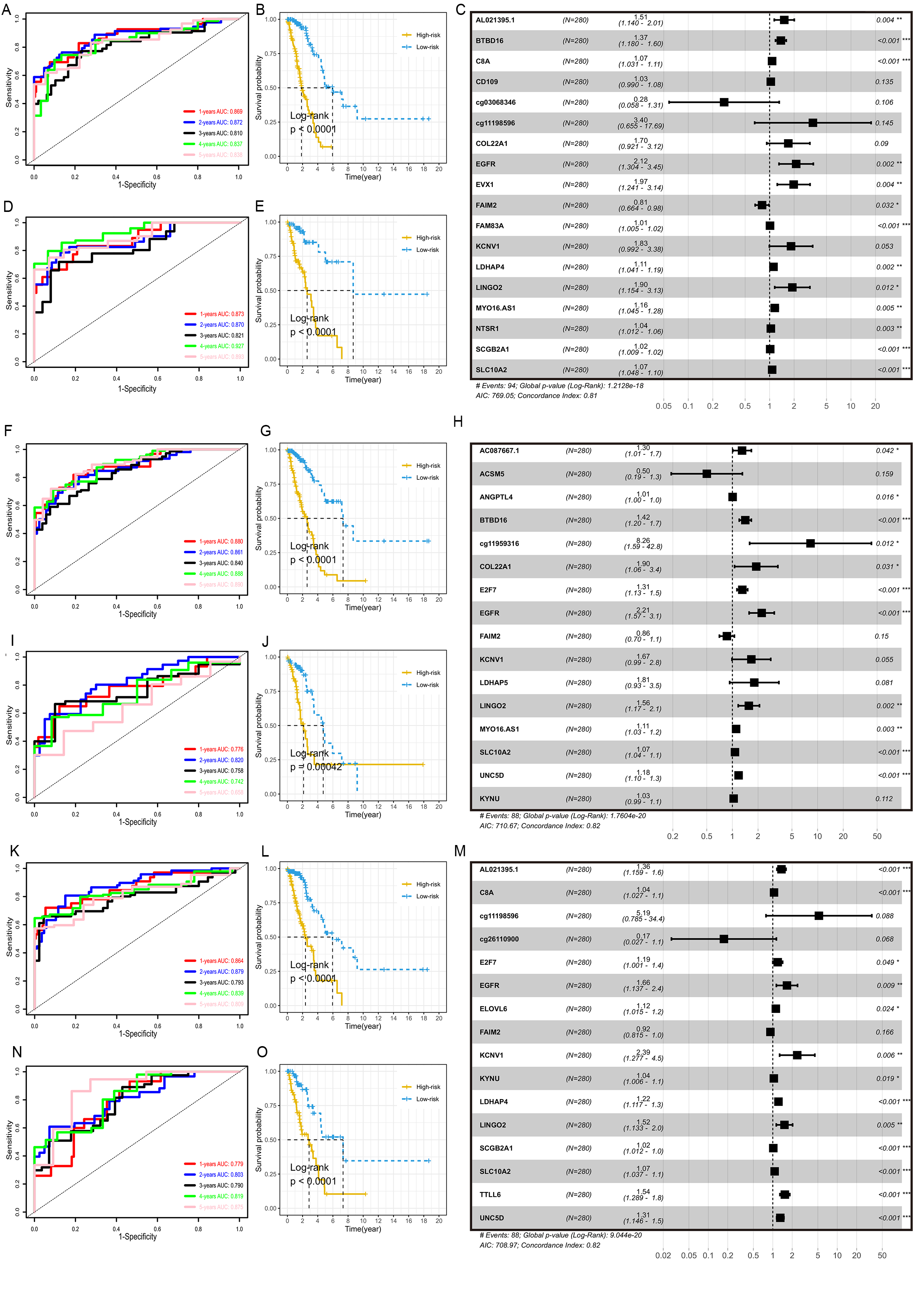

Supplement: Supplementary file 5 — Figure S5. [file JCMM-28-e18032-s004.tif]
